# Digital Droplet PCR for Detection and Quantitation of Hepatitis Delta Virus

**DOI:** 10.14309/ctg.0000000000000509

**Published:** 2022-06-08

**Authors:** Ling Xu, Xiangying Zhang, Yaling Cao, Zihao Fan, Yuan Tian, Huanbin Zou, Yingmin Ma, Zhongping Duan, Feng Ren

**Affiliations:** 1 Beijing Institute of Hepatology, Beijing Youan Hospital, Capital Medical University, Beijing, China.

## Abstract

**INTRODUCTION:**

Hepatitis delta virus (HDV) far exceeds our expected level. There remains a lack of reliable quantitative assays for HDV RNA detection. We sought to develop a new method based on digital droplet polymerase chain reaction (ddPCR) for HDV quantitative detection.

**METHODS:**

With plasmid (pMD19T) containing HDV full genome, we determined the method for ddPCR-based HDV RNA quantification. To compare various assays for HDV detection, 30 cases diagnosed with hepatitis D and 14 controls were examined using enzyme-linked immunosorbent assay, reverse-transcriptase PCR (RT-PCR), and ddPCR. A total of 728 hepatitis B virus–related patients, including 182 patients with chronic hepatitis B, 182 with liver cirrhosis, 182 with hepatocellular carcinoma, and 182 with liver failure, were screened for HDV infection.

**RESULTS:**

The detection limit of ddPCR for HDV is significantly low, with lower limit of detection and lower limit of quantitation of 0.29 IU/mL (95% confidence interval: 1.93 × 10^−3^–1.22 IU/mL) and 8.76 IU/mL (95% confidence interval: 1.83–1.03 × 10^6^ IU/mL), respectively. Among the 44 samples, the enzyme-linked immunosorbent assay detected 30 cases positive, ddPCR reported 24 samples, and RT-PCR reported 10 samples positive for HDV RNA. Moreover, the positive rates of anti-HDV were 1.1%, 3.3%, 2.7%, and 7.1% in patients with chronic hepatitis B, liver cirrhosis, hepatocellular carcinoma, and liver failure, respectively; the detection rates of RT-PCR in HDV RNA were 0%, 16.67%, 15.4%, and 20%, respectively. However, the detection rates of ddPCR were 0%, 33.33%, 30.77%, and 60%, respectively.

**DISCUSSION:**

We establish a high sensitivity and specificity quantitative HDV RNA detection method based on ddPCR. Hepatitis B virus–related end-stage liver diseases, especially liver failure, are associated with a remarkably high rate of HDV infection.

## INTRODUCTION

Hepatitis delta virus (HDV) was found in the nucleus of hepatocytes in 1977 by Italian scholars and is the smallest known human virus (1). HDV is a defective RNA virus, and its proliferation and propagation are dependent on the assistance of hepatitis B virus (HBV) to provide the viral envelope (2). Full-length genomic nucleotide sequencing and phylogenetic analyses have identified 8 genotypes of HDV, with separation among genotypes up to 40% over the full-length sequence. HDV-1, which comprises 4 subgenotypes, is the most prevalent genotype worldwide, and the geographic distributions of genotypes show obvious differences (3). Compared with HBV monoinfection, patients with HDV and HBV coinfection have the most severe form of viral hepatitis. HDV infection significantly accelerates disease progression of chronic hepatitis B, which progresses to cirrhosis within 5 years and to hepatocellular carcinoma within 10 years on average (4,5).

The latest meta-analysis showed that 0.16% of the general population is estimated to be positive for anti-HDV antibodies, with approximately 4.5% of patients infected with HBV being coinfected with HDV worldwide (6). Another recent study estimated that 48–60 million individuals are infected globally (7). In addition, the prevalence of HDV differs significantly among geographic regions. One study conducted from 2011 to 2016 in the United States showed that 42% of adult HBsAg carriers have antibodies against HDV (8). In China, a recent meta-analysis showed an anti-HDV antibody prevalence rate of 2.1% among HBsAg carriers and 0.4% among the general population (6). However, the new cases reported to China CDC were 441, 481, 356, 352, and 187 from 2016 to 2020, which reveals a large discrepancy from the numbers of cases estimated by the prevalence rates (9). Approximately 10% of HBsAg-positive individuals in China are estimated to be infected with HDV, and approximately 90 million individuals in China have HBV infection (10,11). Because of the limited ability to effectively detect HDV, infection with this virus is likely to be a major obstacle for achieving the WHO's goal of eliminating viral hepatitis, including chronic hepatitis B, C, and D, worldwide by 2030.

At present, the limitations of HDV laboratory examinations are one of the important reasons why HDV infection is underestimated. All immunocompetent patients infected with HDV can produce anti-HDV antibodies, including immunoglobulin (Ig) M and IgG. Positivity for anti-HDV IgM indicates HDV replication, whereas IgG suggests a previous HDV infection and persists for many years. Serum HD antigen is detectable only transiently in blood specimens collected early at the onset of HDV infection, before the rise of antibody titers. In clinical laboratories, enzyme-linked immunosorbent assay (ELISA) to detect anti-HDV IgM or IgG is the most common measure to screen for HDV infection. On the one hand, there is a lack of uniform quality standards for kits produced by different manufacturers, and the results of different laboratories are not comparable; on the other hand, test results are inaccurate if the virus infection occurs within the window period and is closely related to the patient's own immune status (12). Although the quantitative microarray antibody capture assay, which has high specificity and sensitivity, is likely an ideal tool for population screening (13), it is not widely used. However, HDV RNA detection is considered the gold standard for diagnosing hepatitis D infection. Recently, real-time reverse-transcriptase polymerase chain reaction (RT-PCR) assays have been used for relatively quantitative detection of HDV, and the sensitivity and accuracy have improved significantly. According to several studies, the quantification limit of a commercially HDV kit based on the RT-PCR assay was reported as 10^2.75^ IU/mL (14); and a dual-targeting RT-PCR assay for HDV RNA detection developed in 2018 showed that the lower limit was 575 IU/mL (15); an HDV RNA quantification assay based on the Cobas TaqMan-based in-house PCR system showed linearity between 3 × 10^2^ and 10^7^ copies/mL (16). However, the detection limit of the RT-PCR assay for HDV is still insufficient for patients with hypoviremia. Moreover, because of the complexity of primers and the probe design, this assay is not well standardized, and results from different laboratories are difficult to compare (17).

The droplet digital PCR (ddPCR) system is based on sample dropletization. After PCR amplification, the concentration of the target molecule can be quantified to 1 copy/μL. In this study, we developed and present a new assay for HDV measurement based on ddPCR that is characterized by improved sensitivity and accuracy compared with RT-PCR. Here, we compare the ELISA, RT-PCR, and ddPCR for HDV RNA detection and explore the prevalence and quantity of HDV in patients with HBV-related liver disease.

## METHODS

### Clinical data

From 2015 to 2020, a total of 772 unique serum samples from HBV-infected patients were collected at Beijing Youan Hospital, Capital Medical University. Among them, the 44 patients enrolled in the study included 30 cases of HBV and HDV coinfection; these patients were positive for anti-HDV IgG or IgM by the ELISA method at least 3 times in the clinical laboratory, and 14 cases of HBV infection alone were used as negative controls. In addition, 728 HBsAg+ serum samples were used for HDV screening, including 182 patients diagnosed with chronic hepatitis B (CHB), 182 diagnosed with HBV-related liver cirrhosis (LC), 182 diagnosed with HBV-related liver failure (LF), and 182 diagnosed with HBV-related hepatocellular carcinoma (HCC). The HBV viral load and HBV serum markers of all enrolled samples were analyzed by the laboratory department of Beijing Youan Hospital. Detailed information on the patients was obtained from medical records. The serum sample from each patient was collected and stored at −80°C until processing. For all samples collected, details were anonymized during subsequent laboratory tests.

The study was approved by the medical ethics committee of Beijing Youan Hospital, Capital Medical University, and written informed consent was obtained from each patient. The procedures followed were in accordance with the ethical standards of the responsible committee on human experimentation and with the 2013 Declaration of Helsinki.

### Enzyme-linked immunosorbent assay

Serum total anti-HDV, anti-HDV IgM, and anti-HDV IgG were determined with ELISAs using commercial kits (abx055820, abx055546, and abx053050; Abbexa, Cambridge, UK) according to the manufacturer's instructions. Briefly, serum samples were diluted at 1:11 with a diluent; the appropriate positive and negative controls were established. Sample and blank wells on the precoated plate were added, and the plate was covered and incubated at 37°C for 30 minutes. Then, the plate was washed 5 times with wash buffer, the detection reagent was added, and the plate was incubated and washed as above. Finally, 50 μL of 3,3′,5,5′-tetramethylbenzidine substrates A and B were added, followed by incubation at 37°C for 10 minutes; 50 μL of stop solution was added to each well, and optical density was measured at 450 nm.

### World Health Organization-HDV international standard

The World Health Organization-HDV international standard (WHO-HDV-IS; Paul-Ehrlich Institute, Langen, Germany) was used to calculate the conversion factor for ddPCR and RT-PCR assays, which is the standard for nucleic acid amplification technique–based assays developed by Paul-Ehrlich Institute. The WHO-HDV-ISs were reconstituted in sterile nuclease-free water for the concentration of 575,000 IU/mL, which was followed by a 10-fold serial dilution. The conversion factors were calculated for transforming the results from copies/reaction to international units (IU/mL) obtained through ddPCR and RT-PCR assays.

### Internal RNA control

To prevent the false-negative results due to inefficient extraction procedures or failures in PCR amplification, the freely circulating glyceraldehyde-3-phosphate dehydrogenase (GAPDH) RNA in serum of patients was used as an internal control. We extracted viral RNAs and other circulating nucleic acids from serum of patients. As the internal RNA control, GAPDH was first tested in extracted RNA pellet by RT-PCR and ddPCR assays. The primers and the probe used for GAPDH amplification were as follows:

Forward: 5′-CCACCCATGGCAAATTCC-3′;
Reverse: 5′-TCGCTCCTGGAAGATGGTG-3′;
Probe: 5′-FAM-TGGCACCGTCAAGGCTGAGAACGT-TAMRA-3′.

### RNA extraction and reverse transcription

Total RNA was extracted using a QIAamp Viral RNA Mini Kit (QIAGEN, Valencia, CA) from 150 μL serum samples according to the manufacturer's instructions. Finally, 50 μL nucleic acid extract was obtained and used for reverse transcription.

RNA was reverse transcribed using the PrimeScript 1st Strand cDNA Synthesis Kit (Invitrogen, Carlsbad, CA) with random primers and oligo(dT) primers. The reaction program was as follows: 30°C, 10 minutes; 42°C, 45 minutes; 95°C, 5 minutes; 70°C, 15 minutes; and 4°C forever. The cDNA product was used for subsequent RT-PCR and ddPCR tests simultaneously.

### Probe and primers

Because HDV has 8 different genotypes, we determined the target sequence within the conserved ribozyme region through the alignment of 8 HDV genotypes. Therefore, the primers and probes set in this assay work for all genotypes of HDV. The primer sequences used are as follows:

Forward: 5′-CTCGGTAATGGCGAATGGGA-3′;
Reverse: 5′-TTCTTTCCTCTTCGGGTCGG-3′;
Probe: 5′-FAM-GCTCTCCCTTAGCCATCCGAG-TAMRA-3′.

### Reverse transcription polymerase chain reaction

The template cDNA, primers, and probes described above were used. The 20-μL reaction contained 10 μL 2 × mix, 0.5 μL of forward primer and reverse primer each, 0.5 μL of the probe, 3 μL of template, and 5.5 μL of reaction buffer. Thermal cycling using an ABI7500 Real-Time PCR Detection system was performed at 94°C for 3 minutes, followed by 35 cycles of 94°C for 30 seconds and 58°C for 45 seconds.

### Droplet digital PCR

All ddPCR procedures were conducted following the manufacturer's instructions for the Droplet Digital PCR System (TargetingOne Biotech. Beijing, China; licensed by China Food and Drug Administration, registration number: 20170025; 20190097; 20192220517). In detail, the TaqMan PCR mixture contained 15 μL of 2x ddPCR Supermix (23003), 1.2 μL of primers (10 μM), 0.6 μL of probe (10 μM), and 2 μL of template, and deionized water was added to a final volume of 30 μL. Then, 30 μL of PCR mixture and 180 μL microdroplet generation oil (10001) are mixed by the microdroplet generation chip to generate water-in-oil droplets with Drop Maker M1. Then, the microdroplet sample was amplified with a T100 Thermal Cycler. The program was as follows: 95°C for 10 minutes (DNA polymerase activation), followed by 40 cycles of 94°C for 30 seconds and 60°C for 1 minute (annealing), and an infinite 4°C hold. Positive and negative controls were included for each test. Subsequently, the amplified products are placed on the droplet digital PCR reader chip, which is quantified by analyzing software Chip Reader (R1 1.0.2). The threshold between positive and negative droplet populations was set manually using per-plate positive and no-template controls as a guide. Finally, HDV cDNA values are reported as copies/reaction.

### Statistical analysis

For ddPCR assay characterization, the basic principle is to divide a sample into 10,000 of different reaction units so that each microdrop unit contains 1 or more copies of templates, each of which amplifies the target molecule, and then, the FAM signal is calculated for each unit. Reaction units with the fluorescent signal at the end of PCR amplification are recorded as “1” and those without fluorescent signals are recorded as “0.” Units with fluorescent signals indicate that they contain at least 1 copy of the target. According to the Poisson probability distribution formula, the data were analyzed with specific software to calculate the concentration of the target.

The coefficient of determination was assessed by linear regression analysis using Prism 8.00 (GraphPad, La Jolla, CA). In addition, lower limit of detection (LLoD) and lower limit of quantitation (LLoQ) were calculated by probit regression analysis with MedCalc software 19.0.4 (MedCalc, Ostend, Belgium), and the lowest concentrations of 50% and 95% positive samples were detected.

Continuous variables are presented as mean ± SDs. A *P* value <0.05 was considered statistically significant.

## RESULTS

### Sensitivity and dynamic range of the ddPCR assay to detect HDV

**Figure 1.**
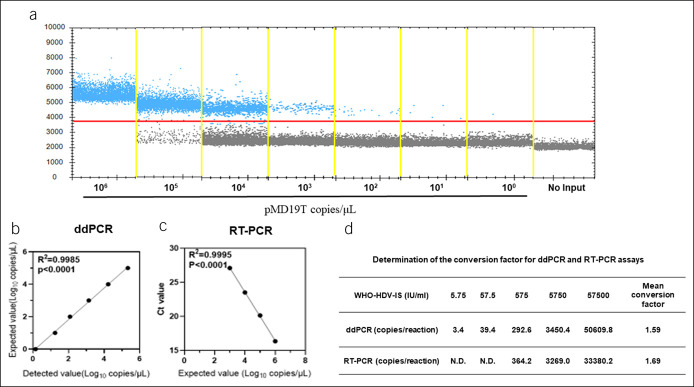
Sensitivity and dynamic range of the ddPCR assay to detect HDV. Data are representative of at least 3 independent experiments. (**a**) The 10-fold serial dilution of pMD19T plasmid is analyzed by the ddPCR; the blue points represent a positive signal. (**b**) Correlation analysis to determine the dynamic range of the ddPCR. The expected values (converted to log10) of pMD19T were plotted on the Y axis vs detected values (converted to log10) on the X axis to perform linear analysis. (**c**) Correlation analysis to determine the dynamic range of RT-PCR. The Ct values reported by the RT-PCR were plotted on the Y axis vs expected values (converted to log10) of pMD19T on the X axis to perform linear analysis. (**d**) Determination of the conversion factors for ddPCR and RT-PCR assays. The 10-fold serial dilutions of WHO-HDV-IS were tested by ddPCR and RT-PCR assays, and the conversion factors were calculated by dividing the given concentrations of standards by the results of ddPCR and RT-PCR assays. ddPCR, digital droplet PCR; HBV, hepatitis B virus; HDV, hepatitis delta virus; RT-PCR, reverse-transcriptase PCR; WHO-HDV-IS, World Health Organization-HDV international standard.

The sensitivity of the ddPCR method for HDV detection was assessed using the plasmid pMD19T containing the HDV full genome, as shown in the Supplementary Materials (see Supplementary Digital Content 1, http://links.lww.com/CTG/A832). A 10-fold serial dilution of the plasmid, ranging from 10^6^ to 10^0^ copies/reaction, was prepared to test the linearity of the ddPCR method using primer and probe sets targeting the HDV common sequence (Figure 1a). Linear regression analysis showed that this method has excellent linear correlation between the detected value and expected value, with R^2^ = 0.9985 (Figure 1b). In addition, RT-PCR was used to detect the above serially diluted plasmid, with a reported range of 10^3^ to 10^6^ copies/reaction. When the target concentration was higher than 10^3^ copies/reaction, RT-PCR displayed good linear correlation, with R^2^ = 0.9995 (Figure 1c).

To transform the results from copies/reaction to international units (IU/mL) for ddPCR and RT-PCR assays, the conversion factors were determined using a series of 10-fold dilutions of the WHO-HDV-IS, which concentration range from 57,500 to 5.75 IU/mL. The estimated conversion factors were 1.59 and 1.69 for ddPCR and RT-PCR methods, respectively (Figure 1b).

Moreover, the reproducibility of ddPCR assay was assessed by intrarun and interrun tests using different positive samples. For intrarun tests, 7 replicates of 3 HDV RNA positive samples (16,160.0 ± 839.5, 2,611.8 ± 156.2, and 2,366.0 ± 118.0 IU/mL) were tested in the same experiment. For interrun tests, 4 individual ddPCR experiments with 2 replicates of 3 HDV RNA-positive samples (8,414.0 ± 238.6, 2,287.4 ± 134.5, and 1,098.5 ± 73.9 IU/mL) were performed. The result shows that mean coefficient of variation (CV) was 5.4% and 5.1% for intrarun and interrun tests, respectively (see Supplementary Table 1, Supplementary Digital Content 1, http://links.lww.com/CTG/A832). These results demonstrated that compared with RT-PCR, ddPCR has a wider detection range, with a lower limit of detection.

### Determining the LLoD and LLoQ of the ddPCR assay for HDV detection

**Figure 2.**
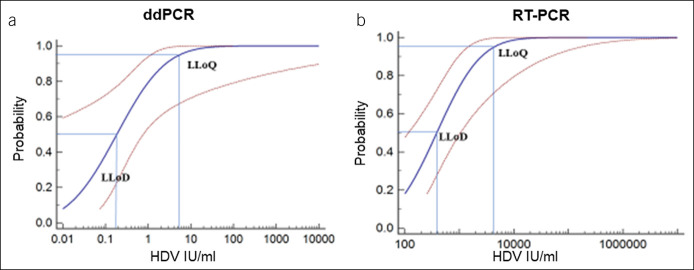
Determining the LLoD and LLoQ of the ddPCR assay for HDV detection. (**a**) The probit analysis sigmoid curve was used to determine the LLoD and LLoQ of the ddPCR. The HDV-positive sample with the determined concentration was diluted in a 10-fold series, each concentration repeated 7 times for ddPCR HDV detection. The concentration corresponding 95% probability on curve represent LLoQ and 50% represent LLoD. (**b**) The probit analysis sigmoid curve was used to determine the LLoD and LLoQ of the RT-PCR. The analytical method was conducted as the same as (**a**). ddPCR, digital droplet PCR; HDV, hepatitis delta virus; LLoD, lower limit of detection; LLoQ, lower limit of quantitation; RT-PCR, reverse-transcriptase PCR.

Next, we performed probit analysis with a sigmoid curve to determine the lower limit of detection (LLoD) and LLoQ of both ddPCR and RT-PCR. First, HDV RNA was extracted from a positive serum sample and then reverse transcribed to cDNA and tested using ddPCR. The sample was serially diluted 10-fold to concentrations from 10^3^ to 10^−2^ IU/mL. Each concentration was tested in 7 replicates, and the LLoD and LLoQ were determined by probit regression with 50% and 95% repeatable probability, respectively. The ddPCR results showed an LLoD of 0.29 IU/mL (95% confidence interval [CI]: 1.93 × 10^−3^–1.22 IU/mL) and LLoQ of 8.76 IU/mL (95% CI: 1.83–1.03 × 10^6^ IU/mL) (Figure 2a). The same method was used to determine the LLoD and LLoQ of RT-PCR, with results of 650.00 IU/mL (95% CI: 196.20–1,769.43 IU/mL) and 7,315.82 IU/mL (95% CI: 2.42 × 10^3^–2.83 × 10^5^ IU/mL), respectively (Figure 2b). Obviously, both ddPCR and RT-PCR were reliable when testing high-concentration samples, but ddPCR performed more precisely when detecting samples with concentrations lower than 10^3^ copies/reaction.

### Specificity of the ddPCR assay for detecting HDV

**Figure 3.**
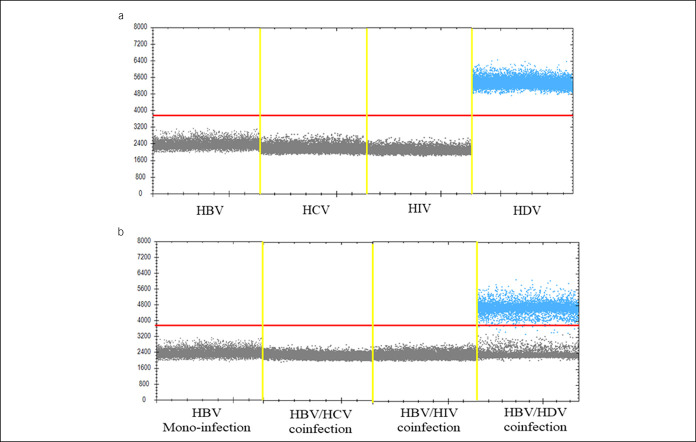
Specificity of the ddPCR assay to detect HDV. (**a**) Plasmids including HBV, HCV, human immunodeficiency virus, and HDV was used to determine the specificity of the ddPCR for HDV detection. The details of plasmids were provided on Supplementary Materials (see Supplementary Digital Content 1, http://links.lww.com/CTG/A832). (**b**) Samples from patients of HBV monoinfection, coinfection of HBV and HCV, coinfection of HBV and human immunodeficiency virus, and coinfection of HBV and HDV to determine the specificity of ddPCR for HDV detection. ddPCR, digital droplet PCR; HBV, hepatitis B virus; HCV, hepatitis B virus; HDV, hepatitis delta virus.

To assess the specificity of ddPCR for HDV detection, we used various kinds of plasmids, including plasmids containing the full-length HBV genome, hepatitis B virus plasmids, human immunodeficiency virus plasmids, and HDV plasmids, to perform 3 independent tests. According to the results, amplification of plasmids other than the HDV plasmid led to no positive signal, which confirms the high specificity of the ddPCR method with the primers/probe set targeting HDV (Figure 3a).

In addition, we evaluated the specificity and effectiveness of ddPCR for HDV detection. We extracted virus nucleic acids from the serum of patients who were clinically confirmed, including cases of monoinfection with HBV, coinfection with HBV and hepatitis B virus, coinfection with HBV and human immunodeficiency virus, and coinfection with HBV and HDV. The above samples were tested using the ddPCR method in parallel, and positive events were found only for patients with a coinfection of HBV and HDV (Figure 3b). In summary, ddPCR for HDV detection is a highly specific method that significantly reduces false-positive results.

### Detection efficiencies of ddPCR, RT-PCR, and ELISA for HDV

In this study, 44 samples were collected from clinical patients, and the clinical information is summarized in Table 1 and Supplementary Table 2 (see Supplementary Digital Content 1, http://links.lww.com/CTG/A832). Thirty samples were from patients clinically diagnosed with hepatitis D, and 14 samples were determined to be HBV infection alone. First, we performed the ELISA to detect anti-HDV IgG, and 30 samples were positive, consistent with the clinical diagnosis. Afterward, viral nucleic acids were extracted from 44 samples simultaneously, and cDNA products were obtained after reverse transcription. The products were first tested GAPDH level by RT-PCR and ddPCR assays, and then HDV RNA was measured.

**Table 1.** Clinical information for 44 confirmed patients

Variables	HBV-related markers						HDV-related markers			GAPDH	
	HBV viral load (IU/mL)	HBsAg	Anti-HBs	HBeAg	Anti-HBe	Anti-HBc	Anti-HDV	RT-PCR (IU/mL)	ddPCR (IU/mL)	RT-PCR (CT value)	ddPCR (copies/reaction)
Patient 1	200	+	−	−	−	+	+	ND	3,544.11	25.03	5,006.3
Patient 2	1,006	+	−	−	−	+	+	28,817.78	66,139.71	24.67	8,993.5
Patient 3	3 × 10^4^	+	−	−	+	+	+	28,716.50	33,891.65	27.51	426.4
Patient 4	4,616	+	−	−	+	+	+	13,553.29	31,762.79	25.82	3,550.6
Patient 5	208	+	−	−	+	+	+	10,659.73	7,093.79	24.19	10,331.5
Patient 6	2,323	+	−	−	+	+	+	ND	1,534.51	26.61	874.1
Patient 7	1.6 × 10^4^	+	−	−	+	+	+	ND	451.40	25.33	3,627.8
Patient 8	2.7 × 10^5^	+	−	+	−	+	+	311,500.80	476,802.84	25.94	3,341.2
Patient 9	3,008	+	−	+	+	+	+	11,846.70	14,297.60	27.28	529.0
Patient 10	7.9 × 10^4^	+	−	+	+	+	+	ND	954.38	26.44	897.2
Patient 11	6,009	+	−	−	+	+	+	ND	523.13	26.15	961.5
Patient 12	3,760	+	−	−	+	+	+	ND	365.73	25.57	4,002.3
Patient 13	600	+	−	−	+	+	+	101,549.07	140,285.70	23.81	13,342.8
Patient 14	2,989	+	−	−	+	+	+	43,318.11	962.11	27.60	447.0
Patient 15	2.1 × 10^4^	+	−	−	+	+	+	ND	477.00	25.88	3,881.2
Patient 16	6.7 × 10^6^	+	−	+	−	+	+	ND	351.39	24.68	8,221.4
Patient 17	5.2 × 10^4^	+	−	+	−	+	+	ND	1,227.48	25.76	4,621.8
Patient 18	3,021	+	−	+	−	+	+	10,140.35	8,872.20	26.61	749.6
Patient 19	6,701	+	−	−	+	+	+	ND	ND	27.67	388.5
Patient 20	880	+	−	−	+	+	+	ND	620.10	24.53	9,001.3
Patient 21	1,554	+	−	−	−	+	+	ND	ND	25.35	4,890.7
Patient 22	990	+	−	−	+	+	+	12,577.15	14,007.90	27.14	525.6
Patient 23	1,228	+	−	−	+	+	+	ND	445.20	26.48	880.5
Patient 24	2.2 × 10^4^	+	−	+	−	+	+	ND	302.10	28.61	322.7
Patient 25	394	+	−	−	+	+	+	ND	ND	27.94	639.6
Patient 26	5.3 × 10^5^	+	−	+	−	+	+	ND	ND	26.79	887.3
Patient 27	7,217	+	−	−	+	+	+	ND	302.42	24.52	8,992.5
Patient 28	209	+	−	−	+	+	+	ND	ND	26.24	922.9
Patient 29	1,020	+	−	−	+	+	+	ND	524.70	27.28	738.5
Patient 30	330	+	−	−	−	+	+	ND	ND	28.31	357.3
Patient 31	3.3 × 10^4^	+	−	+	+	+	−	ND	ND	26.63	922.8
Patient 32	8.1 × 10^6^	+	−	+	−	+	−	ND	ND	24.54	7,993.0
Patient 33	7.3 × 10^7^	+	−	+	−	+	−	ND	ND	23.91	12,607.1
Patient 34	2,001	+	−	−	+	+	−	ND	ND	24.73	8,299.6
Patient 35	9.2 × 10^5^	+	−	+	−	+	−	ND	ND	25.88	3,989.7
Patient 36	1.0 × 10^4^	+	−	+	−	+	−	ND	ND	27.90	625.4
Patient 37	8.2 × 10^4^	+	−	+	−	+	−	ND	ND	28.64	302.9
Patient 38	5.6 × 10^4^	+	−	+	+	+	−	ND	ND	26.51	939.3
Patient 39	6,009	+	−	+	+	+	−	ND	ND	26.23	977.4
Patient 40	2.9 × 10^4^	+	−	+	−	+	−	ND	ND	24.01	9,022.1
Patient 41	5.1 × 10^6^	+	−	+	−	+	−	ND	ND	24.69	8,775.6
Patient 42	3.2 × 10^4^	+	−	+	−	+	−	ND	ND	24.88	7,990.4
Patient 43	377	+	−	−	+	+	−	ND	ND	27.81	677.3
Patient 44	6.2 × 10^5^	+	−	+	−	+	−	ND	ND	26.42	938.5

ddPCR, digital droplet PCR; GAPDH, glyceraldehyde-3-phosphate dehydrogenase; HBV, hepatitis B virus; HDV, hepatitis delta virus; ND, not detected; RT-PCR, reverse-transcriptase PCR.

**Figure 4.**
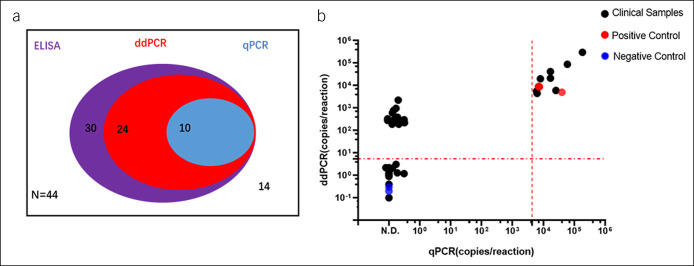
Comparison of the detection efficiency of ddPCR, RT-PCR, and ELISA for HDV. (**a**) The Venn diagram shows the detection number of ddPCR, RT-PCR, and ELISA for HDV in 44 clinical confirmed patients including 30 HDV infection subjects. The purple area shows the number of HDV antibodies positive detected by ELISA; the red area shows the number of HDV RNA detected by the ddPCR; the blue area shows the number of HDV RNA detected by the RT-PCR. (**b**) Comparison of the detection values between the ddPCR and RT-PCR. The measured values of RT-PCR were plotted on the X axis, the vertical dashed line denote the LLoQ of RT-PCR; the measured values of ddPCR were plotted on the Y axis, the parallel dashed line denotes the LLoQ of ddPCR. ddPCR, digital droplet PCR; ELISA, enzyme-linked immunosorbent assay; HDV, hepatitis delta virus; LLoQ, lower limit of quantitation; RT-PCR, reverse-transcriptase PCR.

As shown in Table 1, the internal control and HDV RNA in each sample was detectable by RT-PCR and ddPCR assays. The results of HDV RNA analysis showed that RT-PCR was able to detect only 10 positive samples; ddPCR detected 24 positive samples, 14 more than RT-PCR (Figure 4a,b). Based on our results, positivity for anti-HDV IgG indicates that the patient had a previous infection but that perhaps no virus remained because of the application of antiviral drugs. For the presence of low levels of HDV in patients, the detection efficiency of ddPCR was significantly higher than that of RT-PCR.

### Detection of HDV in patients with HBV-related diseases by ELISA, RT-PCR, and ddPCR

**Figure 5.**
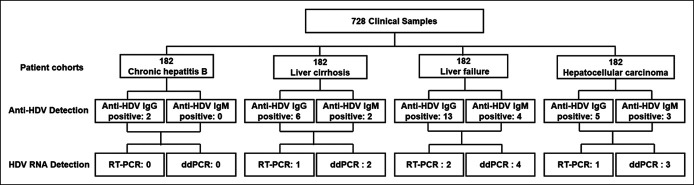
Detection of HDV for patients with HBV-related diseases with ELISA, RT-PCR, and ddPCR. Flow diagram of the patient cohorts of HBV-related disease. ddPCR, digital droplet PCR; ELISA, enzyme-linked immunosorbent assay; HBV, hepatitis B virus; HDV, hepatitis delta virus; RT-PCR, reverse-transcriptase PCR.

A total of 728 samples were examined in this study, including 182 from patients with CHB, 182 from patients with HBV-related LC, 182 from patients with HBV-related LF, and 182 from HBV-related HCC (Figure 5), whose basic information is summarized in Supplementary Table 3 (see Supplementary Digital Content 1, http://links.lww.com/CTG/A832). First, anti-HDV IgG and anti-HDV IgM in these samples were detected using ELISA methods. As shown in Table 2, the results were as follows: 1.1% of patients with CHB were anti-HDV IgG positive; 3.3% of patients with LC were anti-HDV IgG positive, and 1.1% were anti-HDV IgM positive; 2.7% of patients with HCC were anti-HDV IgG positive, and 1.6% were anti-HDV IgM positive; and 7.1% of patients with LF were anti-HDV IgG positive, and 2.2% were anti-HDV IgM positive. Next, total RNA was extracted from the 26 samples that were anti-HDV positive mentioned above, and the internal control and HDV RNA were detected by RT-PCR and ddPCR methods. RT-PCR results showed that no HDV RNA was detected in patients with CHB; 0.55% of LC and HCC cases were positive for HDV RNA and only 0.1% of patients with LF. The ddPCR results were as follows: no HDV RNA positivity in patients with CHB; 1.1% of patients with LC; 1.6% of patients with HCC; and 2.2% of patients with LF. In detail, the 26 patients screened positive for anti-HDV were presented in Supplementary Table 4 (see Supplementary Digital Content 1, http://links.lww.com/CTG/A832), including the positivity of anti-HDV IgG and IgM, HDV RNA values, and GAPDH reported by the RT-PCR and ddPCR. These results indicate that the ddPCR we established is a high-sensitivity and high-accuracy detection method for HDV, especially for HDV RNA less than 10^2^ IU/mL, which is significantly superior to the RT-PCR.

**Table 2.** Detection of HDV for patients with HBV-related diseases

Patient cohorts	Anti-HDV IgG		Anti-HDV IgM		RT-PCR			ddPCR		
	Number	Positive rate (%)	Number	Positive rate (%)	Number	HDV-positive rate in total (%)	RNA-positive rate in HDV infection (%)	Number	HDV-positive rate in total (%)	RNA-positive rate in HDV infection (%)
Chronic hepatitis B (n = 182)	2	1.1	0	—	0	—	—	0	—	—
Liver cirrhosis (n = 182)	6	3.3	2	1.1	1	0.55	16.67	2	1.1	33.33
Liver failure (n = 182)	13	7.1	4	2.2	2	1.1	15.4	4	2.2	30.77
Hepatocellular carcinoma (n = 182)	5	2.7	3	1.6	1	0.55	20	3	1.6	60

ddPCR, digital droplet PCR; HDV, hepatitis delta virus; Ig, immunoglobulin; RT-PCR, reverse-transcriptase PCR.

In addition, we found that HDV prevalence differs among patients with CHB, HBV-related LC, HBV-related LF, and HBV-related HCC. Patients with confirmed CHB showed a relatively low HDV infection rate of 1.1% according to anti-HDV IgG compared with those with LC, HCC, and LF. In patients with LC and HCC, HDV infection rates were 3.3% and 2.7% according to anti-HDV IgG, respectively. In patients with LF, anti-HDV IgG positivity was detected in 7.1%, which indicated that coinfection of HDV and HBV significantly accelerates the progression of liver disease.

## DISCUSSION

It is important to develop an accurate HDV RNA quantitative test, which is critical for HDV diagnosis and guiding treatment response. Several meta-analyses in recent years have reported an infection rate of HDV of approximately 0.8% of the general population and 13.02% of the HBsAg-positive population (7), although the anti–HDV-positive rate increases by 3–4 times among those with liver diseases (6). Currently, there are 8 genotypes of HDV identified by the full-length genomic nucleotide sequences and phylogenetic analyses, and their geographical distribution shows considerable variations. HDV genotype 1 is the most common one all over the world. Genotype 2 is predominantly identified in Asia and has also emerged in Egypt and Iran. Genotype 3 has been reported mainly in South America, and genotype 4 is found in Taiwan and Japan. Finally, genotypes 5–8 are identified only in African patients. Nevertheless, detection of HDV is often neglected clinically. There may be 2 reasons: on the one hand, many clinicians lack awareness of the serious disease consequences caused by HDV infection; on the other hand, there are no standard and accurate methods for detecting HDV infection (18,19). There have been many efforts for HDV detection, including HDV antibody screening by the ELISA (20–22) and quantitative microarray antibody capture assays (13). However, there is a lack of progress in testing HDV RNA quantitatively, and RT-PCR, which is insufficient regarding sensitivity and accuracy, remains the method for confirming the diagnosis and management of patients (23–26). Therefore, a method of HDV RNA quantitation with high sensitivity and specificity for the confirmation of HDV infection and treatment monitoring is urgently needed.

In this study, we developed a new method of detecting HDV RNA based on ddPCR, which enables absolute quantification of the serum virus with excellent sensitivity and high specificity. ddPCR is a very sensitive and reproducible technique that has been used for testing in various fields. Studies have indicated that ddPCR can be used for the detection and quantitation of HBV cccDNA in the liver of individuals with occult HBV infection (27), for the detection of TP53 deletions and point mutations in chronic lymphocytic leukemia (28), and as a more accurate tool for SARS-CoV-2 detection in low viral load specimens (29). The more accurate HDV RNA detection method established in this study significantly improves the diagnostic ability of HDV infection, including confirmation of HDV infection, especially in patients with low viral loads (cases that are underdetermined by the RT-PCR), monitoring the treatment effect of various antiviral drugs, and evaluation of disease progression. ddPCR methods have obvious superiority in HDV RNA detection compared with other available methods. Compared with RT-PCR, our results showed that the sensitivity threshold of these assays was approximately 10^3^ copies/μL, which is significantly lower than the sensitivity of ddPCR at 1 copy/μL. Moreover, the results obtained from different laboratories are often not comparable because of the use of different primer sets and the nonuniformity of the amplified RNA region (30,31). More importantly, commercial and in-house RT-PCR assays in 55% of laboratories often underestimate or fail to quantify HDV viremia, according to the French national quality control study (32). Therefore, considering the limitation of RT-PCR assays for quantitative HDV RNA, the development of more accurate methods for nucleic acids is particularly necessary at present. In addition, detecting HDV-specific IgM or IgG with the ELISA is an indispensable approach, especially for the screening of a large range of HBsAg-positive populations. Nonetheless, the widow period for detection is relatively short (33). Anti-HDV IgM typically appears in serum at 2–3 weeks after the onset of symptoms and disappears by 2 months after acute HDV infection. Therefore, anti-HDV IgG, as a serologic scar, is commonly used for HDV screening because it can persist in serum after the resolution of acute HDV infection and in chronic HDV infection. Furthermore, anti-HDV detection is usually false negative in immunodeficiency patients, such as those with acquired immunodeficiency syndrome. However, confirmation of HDV infection still relies on detectable HDV RNA as a gold standard for HDV diagnosis and management. However, there are pros and cons of ddPCR assays. The advantages of ddPCR for HDV detection include the following: first, it can achieve direct quantification of HDV RNA levels in patients with hepatitis D; second, the ddPCR assay is highly sensitive and specific to detect low levels of HDV RNA; third, the ddPCR assay can achieve dynamic monitoring during HDV antiviral therapy. However, the drawbacks of the ddPCR assay for HDV detection include the following: first, the ddPCR assay requires professional instrumentation to perform in laboratories; second, the cost of this assay is about 26.5 USD for 1 test, which is higher than that of the RT-PCR; third, the time this assay requires is similar to that consumed by the RT-PCR but difficult to achieve high-throughput detection.

In addition, we screened HDV infection in different patient cohorts, including cases of CHB, HBV-related LC, HBV-related HCC, and HBV-related LF. The most striking finding is that the HDV infection rate was as high as 7.1% in patients with HBV-related LF and that it was relatively low in patients with LC or HCC and lowest in patients with CHB. Moreover, we developed RT-PCR and ddPCR for all anti-HDV-positive samples; expectedly, the detection rate of HDV RNA with ddPCR was significantly higher than that of RT-PCR. However, the reasons why samples were positive for anti-HDV but negative for HDV RNA are as follows: (i) the anti-HDV IgG positivity does not indicate active viral replication (ii) and RNA degradation during sample storage. According to a meta-analysis reported in 2020 (6), the pooled proportion with HDV RNA detection was 58.5% (95% CI: 52.4–64.5) in anti–HDV-positive people. Nonetheless, the results strongly indicate that we should conduct early screening for HDV infection in HBsAg-positive individuals, as early detection of HDV infection and timely antiviral intervention can effectively slow the progression of severe liver disease. According to a recent study, there are approximately 120 million people who are positive for HBsAg in China. Based on our results, 7.1% of patients who developed LF from CHB have HDV infection, which is overlooked. Our results have far-reaching implications for public health and institutions related to the development and implementation of appropriate response measures.

In conclusion, we developed an assay based on the ddPCR for HDV detection with high sensitivity and specificity. The assay can quantitatively measure the HDV viral load in patients and is of great significance for guiding the clinical treatment of HDV and monitoring drug efficacy.

## CONFLICTS OF INTEREST

**Guarantor of the article:** Feng Ren, PhD.

**Specific author contributions:** F.R., Z.D., and Y.M. designed the experiments. L.X. and X.Z. performed the experiments and wrote the manuscript. Y.C., Z.F., and Y.T. prepared the samples and collected the data. H.Z. performed statistical analyses. All authors have read and approved the submission of the manuscript.

**Financial support:** This study was supported by grants from the National Natural Science Foundation of China (81770611, 82002243); key projects of Beijing Municipal Education Commission's Science and Technology Plan (KZ202010025035); Capital Health Development Scientific Research Project (2020-1-1151, 2021-1G-2181); Demonstrating application and research of clinical diagnosis and treatment technology in Beijing (Z191100006619096, Z191100006619097); Beijing Talents foundation (2018000021469G289); Beijing Hospitals Authority Youth Programme (QML20201702). The Beijing Municipal Institute of Public Medical Research Development and Reform Pilot Project (JYY2021-10). Talent Cultivation plan of 'Climbing the peak' of Beijing Municipal Hospital Administration (DFL20221503).

**Potential competing interests:** None to report.

**Study Highlights**

**WHAT IS KNOWN**

✓ Chronic infection with both hepatitis B virus (HBV) and hepatitis delta virus (HDV) manifests as the most severe form of viral hepatitis.
✓ The prevalence and disease burden of HDV infection was underestimated because of inadequate screenings for HDV in HBsAg-positive patients.

**WHAT IS NEW HERE**

✓ We established a new method based on the digital droplet PCR for HDV RNA detection with high sensitivity and specificity.
✓ We screened HDV infection in patients with different HBV-related liver diseases and found that liver failure is associated with a remarkably high rate of HDV infection.

## Supplementary Material
