# Supplementary material for: Digital Droplet PCR for Detection and Quantitation of Hepatitis Delta Virus
Source: Clin Transl Gastroenterol. 2022 Jun 8;13(7):e00509. doi: 10.14309/ctg.0000000000000509 (PMC10476728; doi:10.14309/ctg.0000000000000509)
Supplement: Supplementary file 1 [file ct9-13-e00509-s001.docx]

**Supplement Materials**

**Plasmid information**

(1) HDV plasmid: the pMD19T plasmid contains the full-length genome of HDV as follows: ATGGGCCACGGTTCCGACGAGGAGCCGGAGGGGGAGGGGTTGGAGCTCCCGAGGGGGAGTCGAGGTAAAGAGGGGAGGAATCTCGAGAAAGAACTCCCAAGAAGAACCGAGAAGAATCTCAAGAAGAGGAGGTTTCCCAAACGCTGGTGGAGCATCCTGGAAGGGGAAAGAGGAGAAGGATCGGAAGAAAAGAGCGGGCCTCCCGATCCGAGGGGCCCGGACACCTCAGGTTTGGAGGCCTCCGGGCCGAAGGGTTGAGGTACACCGCAGAGGGAGGAATCCACCCGGAGAGAACAGAGAAATCACCTCCAGAGGACCCCTTCAGCGAACAGAGAAGCGCTCTGGGACGTCAGGAGTAAGACCATAGCGATAGGGGGAGATGCTAGGAGTTGGGGGAGACCGAAGCGAGGAGGAAAGCAAAGAAAGCAACGGGGCTAGCGAGTGGGTGTTCCGCCCCCCGGAGGGGACGAGTGAGGCTTATCCCGGGGAACTCGGCGAATCGTCCCCACATAGCAGGATCCCCGGACCCCCTTCCAAAAGGACGAGGGGGGGGGCTTGGAACGTGAGGGGACCAGTGGAGTCCGTGGGAGTTCCCCCCGACCTCCGCTTCTCCACACTCCTTCCCCCCTGAGGGCCCCCCAGGAATGGCGGGACCCCACCCTCGGGGTCCGCGTTCCATCCTTTCTTACCTGATGGCCGGCATGGTCCCAGCCTCCTCGCTGGCGCCGGCTGGGCAACATTCCGAGGGGACCGTCCCTCGGTAATGGCGAATGGGACCCAGAACTCTCTCTAGATTCCCAGAGAGAATCGAGAGAAAACTGGCTCTCCCTTAGCCATCCGAGTGGACGTTCTGTCCTCCTTCGGATGCCCAGGTCGGACCGCGAGGAGGTGGAGATGCCATGCCGACCCGAAGAGGAAAGAAGGACGCGAGACGCGAACCTGTGAGTGGAAACCGCTTTATTCACTGGGGTCGACAACTCTGGGGAGAGAAGGGAGGAGGCGGGGGGGAAGAGTATATCCTATGGGAATCCCTGGTTTCCCCTGATGTCCAGCCCCTCCCCGGTCCGAGAGAAGGGGGACTCCGGGACTCCTTGCATGCTGGGGACGAAGCCGCCCCCGGGCGCTCCCCTCGGACACCTTTCGAGGGGGTTCACACCCCCAACCCGCGGGCCGGCTACTCTTCTTTCCCTTCTCTCGTCTTCCTCGGTCAACCTCCTGAGTTCCTCTTCTTCCTCCTTGCTGAGGCTCTTCCCTCCCGCGGTCAGCTGCTTCTTCTTGTTCTCGAGGGCCTTCCTTCGTCGGTGATCCTGCCTCTCCTTGTCGGTGAATCCTCCCCTGAGAGGCCTCTTCCTAGGTCCGGAGTCTACCTCCATCTGGTCCGTCCGGGCCCTCTTCGCCGGGGGAGCCCCCTCTCCATCCTTTCCTTTTTCCGATTATTCCTTTGATGTTTCCCAGCCAGGGATTGTCGTCCTCAAGTTTCTTGATTTTCTTCTTGGCCTTCCGGAGGTCCCTCTCGAGTTCCTCCACTTCTTTTCTTCCATTCACCCACTGCTCGAGGATCTCTTCTCTTCCTCCCCTCCGGTTTTTCTTCGATTCGGACCGGCTCATCTCGGAGAGGGGGCGGCGTCCTCAGTACTCTTACTCTTTTCTGTAAAAGAGGAGACTGCTGGACTCCCGCCCCAGTCCGAG

(2) HBV plasmid: The plasmid contained the full-length HBV genome was obtained from Sangon Biotech, Shanghai, China, as previously describe[1].

(3) HCV plasmid: The plasmid contained the full-length HCV genome was obtained from Sangon Biotech, Shanghai, China, as previously describe[2].

(4) HIV plasmid: The plasmid contained the full-length HIV genome was obtained from Sangon Biotech, Shanghai, China, as previously describe[3].

**Supplement Table 1.** **The consistency of the ddPCR assay for HDV RNA detection**

**Intra-run test**

|  | **Sample-1#** | **Sample-2#** | **Sample-3#** |
| --- | --- | --- | --- |
| **Replicates 1 (IU/ml)** | **15186.1** | **2403.0** | **2214.6** |
| **Replicates 2 (IU/ml)** | **16002.2** | **2649.9** | **2231.6** |
| **Replicates 3 (IU/ml)** | **16730.5** | **2718.3** | **2441.6** |
| **Replicates 4 (IU/ml)** | **15787.0** | **2617.6** | **2346.7** |
| **Replicates 5 (IU/ml)** | **15549.7** | **2583.9** | **2481.2** |
| **Replicates 6 (IU/ml)** | **16149.3** | **2448.3** | **2331.9** |
| **Replicates 7 (IU/ml)** | **17715.1** | **2861.8** | **2514.3** |
| **Mean value (IU/ml)** | **16160.0** | **2611.8** | **2366.0** |
| **SD** | **839.5** | **156.2** | **118.0** |
| **%CV** | **5.2** | **6.0** | **5.0** |
| **Mean %CV** | **5.4** | | |

**Inter-run test**

|  |  | **Replicates 1 (IU/ml)** | **Replicates 2 (IU/ml)** | **Mean value (IU/ml)** | **SD** | **%CV** | **Mean %CV** |
| --- | --- | --- | --- | --- | --- | --- | --- |
| **Sample-4#** | **TEST1** | **8238.4** | **8884.1** | **8414.0** | **238.6** | **2.8** | **5.1** |
|  | **TEST2** | **8750.0** | **8573.6** |  |  |  |  |
|  | **TEST3** | **8120.3** | **8173.6** |  |  |  |  |
|  | **TEST4** | **8310.0** | **8262.1** |  |  |  |  |
| **Sample-5#** | **TEST1** | **2129.0** | **2346.4** | **2287.4** | **134.5** | **5.9** |  |
|  | **TEST2** | **2321.7** | **2159.9** |  |  |  |  |
|  | **TEST3** | **2194.0** | **2177.0** |  |  |  |  |
|  | **TEST4** | **2384.2** | **2586.8** |  |  |  |  |
| **Sample-6#** | **TEST1** | **1170.7** | **1240.4** | **1098.5** | **73.9** | **6.7** |  |
|  | **TEST2** | **1029.1** | **1059.4** |  |  |  |  |
|  | **TEST3** | **1081.4** | **1027.0** |  |  |  |  |
|  | **TEST4** | **1172.0** | **1008.2** |  |  |  |  |

**Supplement Table 2.** **Demographic data for 44 confirmed patients**

| **Variables** | **HDV positive patients**  **(n=30)** | | **HDV negative patients**  **(n=14)** | **P value** |
| --- | --- | --- | --- | --- |
| **Age, years** | | **72 (67-80)** | **68 (61-72)** | **0.013** |
| **Gender, male/female** | | **18/12** | **6/8** | **>0.05** |
| **HBV viral load, IU/mL** | | **1713.36 (919.4-2507)** | **603.37 (-346.1-1553)** | **>0.05** |
| **HBsAg positivity** | | **30 (100%)** | **14 (100%)** | **>0.05** |
| **Anti-HBs positivity** | | **0 (0%)** | **0 (0%)** | **>0.05** |
| **HBeAg positivity** | | **8 (26.67%)** | **12 (85.71%)** | **0.014** |
| **Anti-HBe positivity** | | **16 (53.33%)** | **10 (71.43%)** | **0.025** |
| **Anti-HBc positivity** | | **30 (100%)** | **14 (100%)** | **>0.05** |

**Supplement Table 3.** **Demographic data for HBV related patient cohorts**

| **Variables** | **Chronic hepatitis B**  **(n=182)** | **Liver cirrhosis**  **(n=182)** | **Liver failure**  **(n=182)** | **Hepatocellular carcinoma**  **(n=182)** | **P value** |
| --- | --- | --- | --- | --- | --- |
| **Age, years** | **45 (28-55)** | **56 (46-68)** | **77 (69-83)** | **65 (61-72)** | **>0.05** |
| **Gender, male/female** | **101/71** | **97/85** | **80/102** | **112/70** | **>0.05** |
| **HBV viral load, IU/mL** | **2.1*10^5^ (1.6*10^3^-6*10^7^)** | **3.7*10^4^ (8.2*10^3^-6.1*10^5^)** | **7.7*10^5^ (3.4*10^4^-5.2*10^6^)** | **1.6*10^3^ (4.7*10^2^-2.2*10^5^)** | **>0.05** |
| **HBsAg positivity** | **182 (100%)** | **182(100%)** | **182(100%)** | **182 (100%)** | **>0.05** |
| **Anti-HBs positivity** | **0 (0%)** | **0 (0%)** | **0(0%)** | **0 (0%)** | **>0.05** |
| **HBeAg positivity** | **167 (91.76%)** | **158 (86.81%)** | **163 (89.56%)** | **149 (81.87%)** | **>0.05** |
| **Anti-HBe positivity** | **132 (72.53%)** | **112 (61.54%)** | **108 (59.34%)** | **119 (65.38%)** | **>0.05** |
| **Anti-HBc positivity** | **182 (100%)** | **182 (100%)** | **182 (100%)** | **182 (100%)** | **>0.05** |

**Supplement table 4. The results of HDV-positive patients in different patient cohorts**

|  | **Anti-HDV** | | **RT-PCR** | | **ddPCR (IU/ml)** | **GAPDH** | |
| --- | --- | --- | --- | --- | --- | --- | --- |
|  | **Anti-**  **HDV IgG** | **Anti-**  **HDV IgM** | **CT value** | **HDV RNA**  **concentration (IU/ml)** |  | **RT-PCR**  **(CT value)** | **ddPCR**  **(copies/reaction)** |
| **CHB-33#** | **+** | **-** | **-** | **-** | **-** | **24.60** | **8789.2** |
| **CHB-89#** | **+** | **-** | **-** | **-** | **-** | **27.26** | **619.4** |
| **LC-18#** | **+** | **-** |  |  |  | **27.09** | **664.5** |
| **LC-22#** | **+** | **+** |  |  | **1342.60** | **24.04** | **9298.0** |
| **LC-40#** | **+** | **-** |  |  |  | **25.93** | **3556.1** |
| **LC-49#** | **+** | **-** |  |  |  | **27.25** | **606.7** |
| **LC-86#** | **+** | **+** | **29.598** | **12990.86** | **13967.04** | **26.33** | **922.1** |
| **LC-104#** | **+** | **-** |  |  |  | **27.42** | **670.6** |
| **LF-16#** | **+** | **+** |  |  | **1158.80** | **25.16** | **4008.9** |
| **LF-21#** | **+** | **-** |  |  |  | **25.87** | **3858.2** |
| **LF-24#** | **+** | **-** |  |  |  | **24.99** | **4702.6** |
| **LF-42#** | **+** | **-** |  |  |  | **27.23** | **651.9** |
| **LF-49#** | **+** | **-** |  |  |  | **26.83** | **829.5** |
| **LF-52#** | **+** | **+** |  |  | **1734.69** | **27.01** | **703.5** |
| **LF-58#** | **+** | **+** | **29.505** | **13721.72** | **7023.98** | **27.39** | **599.3** |
| **LF-64#** | **+** | **-** |  |  |  | **25.54** | **3780.0** |
| **LF-67#** | **+** | **-** |  |  |  | **25.91** | **3321.9** |
| **LF-88#** | **+** | **-** |  |  |  | **26.87** | **893.3** |
| **LF-121#** | **+** | **-** |  |  |  | **24.47** | **8277.4** |
| **LF-135#** | **+** | **+** | **29.660** | **12525.38** | **9079.70** | **26.16** | **1002.9** |
| **LF-157#** | **+** | **-** |  |  |  | **25.30** | **4333.0** |
| **HCC-40#** | **+** | **+** |  |  | **1089.47** | **28.27** | **287.5** |
| **HCC-58#** | **+** | **+** |  |  | **1676.02** | **26.56** | **934.2** |
| **HCC-82#** | **+** | **+** | **28.859** | **20069.02** | **17167.71** | **26.48** | **996.9** |
| **HCC-107#** | **+** | **-** |  |  |  | **27.66** | **492.7** |
| **HCC-172#** | **+** | **-** |  |  |  | **25.84** | **3007.4** |

**Reference**

[1] S. Wang, H. Li, Z. Kou, F. Ren, et al. Highly sensitive and specific detection of hepatitis B virus DNA and drug resistance mutations utilizing the PCR-based CRISPR-Cas13a system, Clin Microbiol Infect, 27 (2021) 443-450.

[2] N.J. Cho, E.A. Pham, R.J. Hagey, et al. Reconstitution and Functional Analysis of a Full-Length Hepatitis C Virus NS5B Polymerase on a Supported Lipid Bilayer, ACS Cent Sci, 2 (2016) 456-466.

[3] E. Malatinkova, M. Kiselinova, P. Bonczkowski, et al. Accurate quantification of episomal HIV-1 two-long terminal repeat circles by use of optimized DNA isolation and droplet digital PCR, J Clin Microbiol, 53 (2015) 699-701.
